# Diagnosis of Subclinical Keratoconus Based on Machine Learning Techniques

**DOI:** 10.3390/jcm10184281

**Published:** 2021-09-21

**Authors:** Gracia Castro-Luna, Diana Jiménez-Rodríguez, Ana Belén Castaño-Fernández, Antonio Pérez-Rueda

**Affiliations:** 1 Department of Nursing, Physiotherapy and Medicine, University of Almería, 04120 Almería, Spain; djr239@ual.es; 2 Department of Mathematics, University of Almería, 04120 Almería, Spain; acf583@ual.es; 3 Department of Cornea, Hospital of Torrecardenas, 04120 Almería, Spain; a.perezrueda.oft@gmail.com

**Keywords:** subclinical keratoconus, deep learning, corneal topography, random forest

## Abstract

(1) Background: Keratoconus is a non-inflammatory corneal disease characterized by gradual thinning of the stroma, resulting in irreversible visual quality and quantity decline. Early detection of keratoconus and subsequent prevention of possible risks are crucial factors in its progression. Random forest is a machine learning technique for classification based on the construction of thousands of decision trees. The aim of this study was to use the random forest technique in the classification and prediction of subclinical keratoconus, considering the metrics proposed by Pentacam and Corvis. (2) Methods: The design was a retrospective cross-sectional study. A total of 81 eyes of 81 patients were enrolled: sixty-one eyes with healthy corneas and twenty patients with subclinical keratoconus (SCKC): This initial stage includes patients with the following conditions: (1) minor topographic signs of keratoconus and suspicious topographic findings (mild asymmetric bow tie, with or without deviation; (2) average K (mean corneal curvature) < 46, 5 D; (3) minimum corneal thickness (ECM) > 490 μm; (4) no slit lamp found; and (5) contralateral clinical keratoconus of the eye. Pentacam topographic and Corvis biomechanical variables were collected. Decision tree and random forest were used as machine learning techniques for classifications. Random forest performed a ranking of the most critical variables in classification. (3) Results: The essential variable was SP A1 (stiffness parameter A1), followed by A2 time, posterior coma 0°, A2 velocity and peak distance. The model efficiently predicted all patients with subclinical keratoconus (Sp = 93%) and was also a good model for classifying healthy cases (Sen = 86%). The overall accuracy rate of the model was 89%. (4) Conclusions: The random forest model was a good model for classifying subclinical keratoconus. The SP A1 variable was the most critical determinant in classifying and identifying subclinical keratoconus, followed by A2 time.

## 1. Introduction

Keratoconus is a non-inflammatory corneal disease characterized by gradual thinning of the stroma leading to protrusions and irregular astigmatism, which are challenging to correct optically, resulting in irreversible visual quality and quantity decline [1]. Usually, they appear in adolescence and progress until the fourth decade [2]. Although several theories and genetic factors have been proposed [3], the etiology has not yet been elucidated. Currently, treatments can prevent keratoconus progressions, such as intracorneal ring implants and cross-linking [4,5]. Therefore, early detection of keratoconus and subsequent prevention of possible risks are crucial factors in its progression [5].

Early detection also helps us understand the natural course of keratoconus [5,6]. Advanced keratoconus can be diagnosed by classic clinical signs (Munson’s sign, Vogt’s striae, Fleischer ring, etc.) on slit-lamp examination. However, they do not exist in the early stage of keratoconus [7]. Computerized corneal video corneal topography is the most widely used tool to detect the corneal topography pattern and corneal parameters of keratoconus, which can be used for early detection and can distinguish its natural and therapeutic process [8]. Corneal topographical characteristics distinguish keratoconus from normal eyes, such as increased corneal refractive power, inferior-superior (I-S) corneal refractive asymmetry, and steeper radial axis tilt [9]. However, the large number of indicators and complexity provided by video corneal imaging pose clinical challenges for ophthalmologists. [10] Recently, deep learning (DL) has been proven to achieve higher accuracy compared to traditional techniques. Deep learning (DL) is mainly used in medical image analysis and datasets where the DL system shows solid diagnostic using fundus photographs and optical coherence tomography (OCT), [11] for detecting diabetic retinopathy (DR), [12] glaucoma, [13] age-related macular degeneration (AMD) [14] and retinopathy of prematurity (ROP). [15]. The aim has been to use the random forest technique in the classification and prediction of subclinical keratoconus, considering the metrics proposed by Pentacam and Corvis.

## 2. Materials and Methods

This retrospective cross-sectional study was conducted at the Ophthalmology Department of Torre Cardenas University Hospital. It was approved by the Almeria Research Ethics Committee (CEI/CEIm) of Torre Cardenas Hospital. The committee’s reference number is as follows: 19/2019 Helsinki Principles of the Declaration. The design was a cross-sectional study to evaluate the aberration measurement and biomechanical variables of SCKC, using a rotating Scheimpflug camera (Pentacam) to acquire data and the Corvis ST device. Between January 2020 and January 2021, patients with SCKC and healthy corneas were recruited from the ophthalmology service of Torrecárdenas Hospital in Almería, Spain. They are collected from Pentacam and Corvis ST clinical databases. The author declares that there were no conflicts of interest with the manufacturers of Pentacam or Corvis ST.

A total of 81 eyes of 81 patients were enrolled, and the distribution is as follows:

- Sixty-one eyes with healthy corneas. They had the following characteristics: (1) normal morphology, (2) negative keratoconus morphology, (3) no history of eye diseases. Each patient included only one eye;
- Twenty patients with subclinical keratoconus (SCKC): This initial stage included patients with the following conditions: (1) minor topographic signs of keratoconus and suspicious topographic findings (mild asymmetric bow tie, with or without deviation; (2) average K (mean corneal curvature) <46, 5 D; (3) minimum corneal thickness (ECM)> 490 μm; (4) no slit lamp found (no central thinning, Fleischer ring or Vogt pattern); (5) contralateral clinical keratoconus of the eye.

The exclusion criteria applied are ocular or systemic diseases and any ocular surgery, including intracorneal ring segments and corneal collagen cross-linking.

### 2.1. Patient Examination

The patient was examined by the same trained investigator (A.P.R). UCVA and BSCVA were collected using the Snellen graph and logMAR graph. We used an automatic refractometer (KR8900, Topcon, Japan), slit-lamp and fundus to check the eye.

Corneal topography was performed in all patients, under the same light conditions and at a central pupillary diameter of 6.0 mm. Patients with soft contact lenses did not wear them on the examined eye (one for the patient) for three weeks and rigid gas permeable lenses for at least five weeks before testing. Topography was performed with a rotating chamber Scheimpflug (Pentacam HR, Oculus Optikgeräte, Wetzlar, Germany) and biomechanics with Corvis ST.

The following Pentacam variables were collected: topographic variables of the anterior surface of the cornea, the flattest keratometry curvature (K1) and its axis (K1 axis), the steepest keratometry curvature (K2) and its axis (K2 axis), the mean keratometry curvature (Km), the maximum curvature power at the front of the cornea (KMAX), and the asphericity coefficient describing the corneal shape factor (Q). Topographic variables of the posterior surface were collected: the flatter curvature (K1) and its axis (K1 axis), the steeper curvature (K2) and its axis (K2 axis), the mean curvature (Km) and the asphericity (Q). Pachymetric variables: central corneal thickness (CCT), minimum corneal thickness (MCT) with its coordinates (x, y). Related to corneal aberrometry: the mean square of total aberrations (total RMS), the mean square of higher-order aberrations (HOA RMS) which were calculated up to Zernike’s third order for a pupil diameter of 6. 0-mm pupil diameter, astigmatism at 0° (Z22) and 45° (Z2-2), anterior horizontal coma at 0°, posterior horizontal coma at 0°, total horizontal corneal coma at 0° (Z3¹), anterior vertical coma at 90°, posterior vertical coma at 90°, total vertical corneal coma at 90° (Z3-1), trefoil at 0° (Z3-3), trefoil at 30° (Z33), tetrafoil at 0° (Z44), tetrafoil at 22 5° (Z4-4) and spherical aberration (Z40). In addition, Belin/Ambrósio Enhanced Ectasia Display (BAD-D) was included.

Corvis ST variables were: velocity when the air pulse is on (time A1, length A1 and velocity A1) and off (time A2, length A2 and velocity A2), time of highest concavity (time HC), maximum deformation amplitude (DA max), peak distance (PD) and radius of curvature (RHC) at the highest concavity (HC). Vinciguerra indexes: maximum deformation amplitude radius at 2 and 1 mm, integrated radius, relative thickness of Ambrósio in the horizontal profile (ARTh), stiffness parameter at the first flattening (SP-A1) and corneal biomechanical index (CBI). Central corneal thickness (CCT) was calculated using a horizontal Scheimpflug image at the apex. Intraocular pressure was calculated based on the time of the first flattening. It was expressed as bPIO.

### 2.2. Database Cleaning

The steps to depurate the database were:

- Normalization of N (0,1) variables: this step was not necessary in random forest and decision trees; the explanatory variables were not normalized in these methodologies. Other methodologies such as support vector machines, k-neighbors did require such normalization;
- Elimination of variables with marginal variance; this type of variable does not help to build the model;
- Elimination of variables with high correlation since highly correlated independent variables contribute noise and can lead the researcher to erroneous conclusions;
- Elimination of linear dependencies.

### 2.3. Techniques for Working with Unbalanced Data

Mainly, there were two techniques for working with unbalanced data, and both undergo resampling.

- Undersampling: This involves the underrepresentation of the most frequent class or group. In this case, it was the NORMAL class. The NORMAL sample number decreased from 61 to 58 observations;
- Oversampling: Passes through the overrepresentation of the less frequent class or group. In this case, it was the SCKC class. The SCKC sample number boosted from 20 to 54 observations avoiding unbalanced data.

### 2.4. Decision Tree

A decision tree is a predictive model used in various fields of science and social science. The tree starts from a node and branches according to a series of questions and their respective answers.

### 2.5. Random Forest Algorithm

Every time a different partition of the data set is performed, a slightly different classification tree will be obtained. Random forest algorithm is used to control this problem. Random forest is a machine learning technique for classification, based on the construction of thousands of decision trees during the training period of the model and whose answer in each tree is the class with the highest mode value. The random forest methodology protects against the problem of overfitting.

#### 2.5.1. Importance of Variables in the Ranking

Random Forest performs a ranking of the most critical variables in classification problems.

The ranking is calculated based on an importance score.

- Mean decrease accuracy: This shows the amount of accuracy the model loses when excluding each variable. The more accuracy is lost, the more influential the variable is in the ranking;
- Mean decrease in Gini: This coefficient measures how a variable contributes to the homogeneity of the nodes and leaves in the resulting model.

The higher the values of these coefficients, the more influential the variable is in this model.

In this project, we worked with the mean decrease accuracy.

#### 2.5.2. Fold Cross-Validation

This method requires separating the data set into 10 subsets. A subset is selected and kept out of the data set, using the remaining subsets to generate the model. The subset that was isolated and separated will be used to test the model further. This process is repeated ten times and was applied exclusively to the train set to extract the best model parameters and then tested only once on the test set

#### 2.5.3. Confusion Matrix and Metrics

The results were defined in a confusion matrix in which the cases of each class predicted by the algorithm are presented in the rows and the columns the cases for each class. The correctly classified cases are placed on the diagonal of the confusion matrix. The metrics of the confusion matrix are shown below as true positives (TP), true negatives (TN), false positives (FP) and false negatives (FN). Accuracy: proportion of the total number of observations that were classified by the algorithm correctly.

Precision: (TP + TN)/(TP + TN + FP + FN)—proportion of predictions as a positive class that is positive.

- Recall/sensitivity: TP/(TP + FN)—proportion of all positive cases correctly identified as positive by the algorithm;
- Specificity: TN/(TN + FP)—proportion of all negative cases correctly predicted as negative by the algorithm;
- F1-score: combines the precision and recall metrics into a single measure. It is the harmonic mean of both metrics.

(AUROC): the area under the curve. The cumulative distribution function of sensitivity on the y-axis versus the cumulative distribution function of (1-specificity) on the x-axis.

All statistical analyses were performed with R.3.5.1

## 3. Results

### 3.1. Descriptive Statistics

Table 1 and Table 2 show demographics and clinical and biomechanical differences between normal and subclinical keratoconus

### 3.2. Database Cleaning

The highly correlated variables have been eliminated. The highly correlated variables are:

[1] “KMAX” “Km”

[3] “Coma cornea 90°” “Coma ant 90°”

[5] “Coma post 90°” “K2”

[7] “Km p” “K1”

[9] “Integrated Radius [mm-1]” “BCVA”

[11] “K2 Axis” “A1 Deflection Length [mm]”

[13] “Coma ant 0°” “Coma cornea 0°”

[15] “DA Ratio Max (2 mm)”

Therefore, a model including the following independent variables is constructed:

- Demographic variables: age;
- General examination data: LogMAR;
- General topographic indices: K1 Axis, K1 *p*, K1 Axis *p*, K2 *p*, K2 Axis *p*, Qp, CCT;
- Aberrometric topographic indices measured by Pentacam: Total RMS, Ast 0°, Ast 45°, Coma post 0°, Trefoil 0°, Trefoil 30°, Tetrafoil 0°, Tetrafoil 22.5°, Spherical Aberration;
- Biomechanical indices measured by Corvis: A1 Deflection Length, A2 Time, A2 Deflection Length, A2 Velocity, HC Time, PeakDist, Radius, DA Ratio Max, SP A1.

### 3.3. Generation of the New Database

A random and balanced sample was generated from the original one, using the oversampling technique. The final sample size was 112 observations, of which 58 cases corresponded to healthy patients and 54 to patients with subclinical keratoconus.

### 3.4. Decision Trees

The proposed model makes the following decisions: Is SP A1 (biometric index measured by Corvis) greater than or equal to 102? If the answer is yes, then the model classifies the patient directly into a group of patients who do not have subclinical keratoconus. If the answer is no, then the model classifies the patient into the group of patients with subclinical keratoconus.

**Figure 1:**
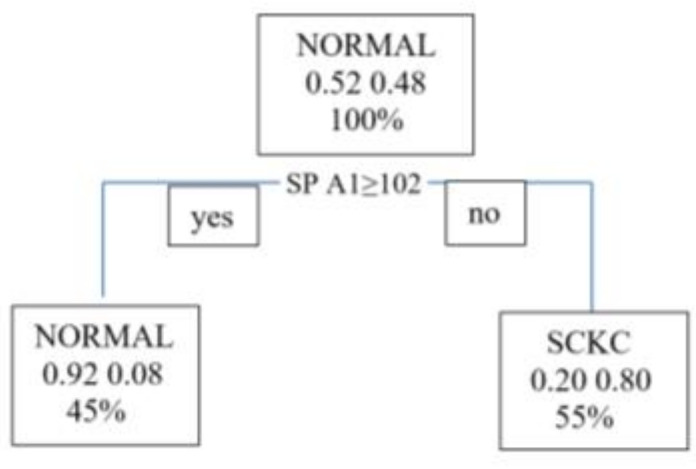
Decision tree. SPA1= Stiffness Parameter at first Applanation; SCKC=Subclinical Keratoconus.

It appears as a simple model in which only one variable (SP A1) influences. The success rate of the model shown in Figure 1 is 71.4%.

We move on to the model validation phase. Table 3 shows that the model efficiently predicts all patients with subclinical keratoconus (Sp = 93%), but it is not excellent at correctly classifying healthy cases (Sen = 50%) (see Table 4). The software (R 3.5.1version) has taken the NORMAL class as positive.

### 3.5. Random Forest

As explained in the previous section, each time you partition the data set differently, you will get a slightly different classification tree. Thus, the way to choose a model is to generate multiple trees and compare them. That is what the random forest algorithm does. The idea of a random forest is to relate the trees that are generated by performing each partition. Then, afterward, to reduce the variance of the trees by averaging them. Averaging the trees helps to reduce dispersion and helps to improve performance. Finally, mention that it avoids overfitting.

#### 3.5.1. Number of Random Variables as Candidates in Each Branch (Mtry)

**Figure 2:**
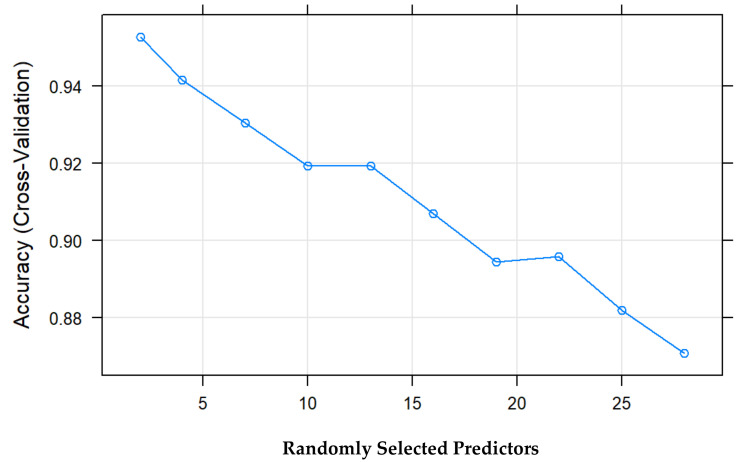
Percentage of success vs. predictors of the model.

Another essential difference added by random forest is that only a random subset of explanatory variables is considered each time a partition is performed. In contrast, in decision trees, all explanatory variables are incorporated. In this case, the best model uses two randomly selected explanatory variables for each branch. The best model is selected based on the model’s percentage of correctness (accuracy) (see Figure 2).

#### 3.5.2. Number of Trees in the Forest (Ntree)

The number of trees used in the model is 500.

#### 3.5.3. Importance of Variables in rf

**Figure 3:**
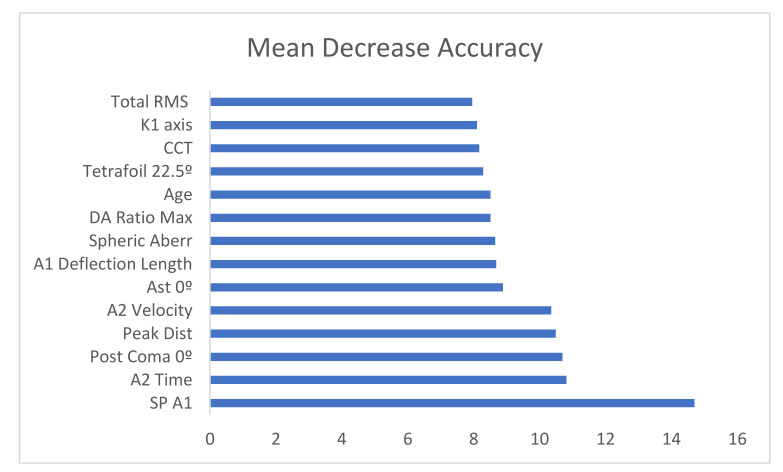
Bar chart with the importance of the variables from highest to lowest.Total RMS = Total Root minimum square, CCT = Central corneal thickness, DA Ratio Max = Deformation amplitude ratio maximum, SP A1 = Stiffness parameter A1.

The importance of variables as a function of mean decrease accuracy is shown in Figure 3.

Figure 3 shows that the essential variable is SP A1, Stiffness parameter A1, which coincides with the tree shown in Figure 1, followed by A2 velocity, closely followed by PeakDist.

Table 5 shows that the model efficiently predicts all patients with subclinical keratoconus (Sp = 93%) and is a good model for classifying healthy cases (Sen = 86%). The overall accuracy rate of the model is 89% (Table 6). The software (R 3.5.1 version) has taken the normal class as positive.

## 4. Discussion

This work aimed to use the random forest technique in the classification and prediction of subclinical keratoconus, considering the metrics proposed by Pentacam and Corvis. Traditional predictive methods generate global models in which a single equation applies to the entire sample space. When the use case involves multiple predictors, which interact in a complex and nonlinear way, it is not easy to find a single global model that can reflect the relationship between the variables. Tree-based statistical and machine learning methods encompass a set of non-parametric supervised techniques that manage to segment the space of predictors into simple regions, within which it is easier to handle interactions. It is this feature that gives them much of their potential.

Tree-based methods have become one of the benchmarks in the predictive field due to the good results they generate in a wide variety of problems. Throughout this paper, we explore how random forest models are built and predicted. Since the fundamental element of a random forest model is the decision trees, it is essential to understand how the latter work. The main advantages are that they can automatically select predictors and apply them to regression and classification problems. Trees can, in theory, handle both numerical and categorical predictors without creating dummy or one-hot-encoding variables. In practice, this depends on each library’s implementation of the algorithm.

Since these are non-parametric methods, no specific distribution is required. In general, they require much less data cleaning and pre-processing compared to other statistical learning methods. Outliers do not significantly influence them. Suppose for any observation, the value of a predictor is not available, despite not reaching any terminal node. In that case, a prediction can be achieved using all observations belonging to the last node reached. The accuracy of the prediction will be reduced, but at least it can be obtained. They are useful in data exploration; they quickly and efficiently identify the most critical variables (predictors). They have good scalability; they can be applied to data sets with a large number of observations.

There are disadvantages: when combining multiple trees, the interpretability of single-tree models is lost. When handling continuous predictors, they lose certain information when categorizing them when splitting the nodes.

As described below, the creation of tree branches is achieved by the recursive binary splitting algorithm. This algorithm identifies and evaluates the possible splits of each predictor according to a given measure (RSS, Gini, entropy, etc.). Continuous or qualitative predictors with many levels are more likely to contain a certain optimal cut-off point by chance; thus, they are usually favored in creating the trees.

The main limitation of the decision tree is that each time you partition the data set differently, you will obtain a slightly different classification tree when using decision trees. Thus, the way to choose a model is actually to generate multiple trees and compare them. It is how the random forest algorithm works. The idea of a random forest is to relate the trees that are generated by doing each partition. Then, reduce the variance of the trees by averaging them. Averaging the trees helps to reduce dispersion and also helps to improve performance. Finally, random forest avoids overfitting.

Regarding the diagnosis of subclinical keratoconus, several authors have used several methods for the automatic diagnosis of ectatic corneal disorders using corneal topography, such as discriminant analysis [6], and subsequently, a neural network approach [16]. These approaches achieved an overall sensitivity of 94.1% and an overall specificity of 97.6% (98.6% for keratoconus only) in the test set. [17] Hwang et al. [18] used multivariate logistic regression analysis to extract available variables from slit-scanning tomography and spectral-domain OCT for clinically normal fellow eyes of patients with highly asymmetric keratoconus. Our team has published several articles on the early diagnosis of keratoconus based on multivariate regression models [19,20].

A large number of studies have been performed with artificial intelligence for keratoconus diagnosis. Thus, Arbelaez et al. [21] performed a study based on support vector machines (SVM) with Sirius obtaining sensitivity 92%, specificity 97.7%, accuracy 97.3% and precision 78.80% to differentiate subclinical keratoconus and normal corneas. Other authors, such as Smadja et al. [22], used decision trees obtaining results after pruning of sensitivity 90.0% and specificity 86.0% to differentiate subclinical keratoconus and normal corneas. Ambrósio et al. [23] used a support vector machine (SVM) and random forest method and obtained a sensitivity of 90.4%, a specificity of 96%, and an AUC of 0.96 for the use of the TBI (tomographic biomechanical index) parameter. Kovács et al. [24] gave great importance to the height decentration index (HDI). They analyzed the patients’ multilayer perceptron in their database, obtaining sensitivity 90.00%, specificity 90.00% and AUC 0.96 to differentiate subclinical and normal keratoconus. In 2019, Castro-Luna et al. [25] used Bayesian classifiers, obtaining a sensitivity, specificity, precision and accuracy of 100% to differentiate normal corneas and keratoconus. Issarti et al. [26] developed a mathematical model called CAD (computer-aided diagnosis) for early keratoconus detection using feedforward neural network combined with Grossberg-Runge-Kutta architecture (feedforward neural network and a Grossberg-Runge Kutta architecture). The results of the CAD model were sensitivity 97.78%, specificity 95.56%, accuracy 96.56% and precision 95.65%. It was compared with the TKC (topographical keratoconus classification) index, the BAD-D (Belin/Ambrosio deviation) index and the CMM-FFN (feedforward neural network combined with mathematical model) model. Xie et al. [27] created a deep learning model named Pentacam inception ResNet V2 screening system (PIRSS) by taking anterior and posterior elevation and corneal thickness data. The model achieved a sensitivity of 92.00%, specificity of 99.10%, accuracy of 98.00% and an AUC of 1.00. Ruiz Hidalgo et al. [28] created a model through support vector machines (SVM). The results obtained when comparing normals with early stages of keratoconus were sensitivity 79.1%, specificity 97.9%, precision 93.0%, accuracy 93.1%, and an AUC of 0.92. Lavric et al. [29] have performed a keratoconus detection algorithm using convolutional neural networks (CNNs) named Keratodetect, obtaining an accuracy of 99.33%.

Several reviews have been performed in the literature about artificial intelligence studies in keratoconus. [30,31].

In this study, a model has been made with the tomographic data through a random forest (RF) that efficiently predicts all those patients with subclinical keratoconus (Sp = 93%), and it is also a good model for classifying healthy cases (Sen = 86%). The overall accuracy rate of the model is 89%. In the study by Lopes et al. [32], the random forest provided the highest accuracy among AI models with a sensitivity of 100% for clinical ectasia. It was named Pentacam random forest index (PRFI). The PRFI had an area under the curve (AUC) of 0.992 (sensitivity = 94.2%, specificity = 98.8% and cutoff point = 0.216), statistically superior to the Belin-Ambrósio deviation (BAD-D; AUC = 0.960, sensitivity = 87.3% and specificity = 97.5%). In our case, for a k = 10 (CV), the random forest model proposed in this work has proven to be an excellent model for the classification of subclinical keratoconus (the accuracy rate is over 89%). The model has 500 decision trees and uses only two random variables in each partition. The SP variable A1 is the most important determinant variable in classifying and identifying subclinical keratoconus, followed by A2 Time. Peris-Martinez et al. [33] published that A2 speed was the best parameter to diagnose patients with subclinical keratoconus. In a recent study, Ren et al. [34] demonstrated that ARTh and SP-A1 variables were lower in eyes with subclinical keratoconus than in normal eyes.

Stiffness parameters are defined as resultant pressure at inward applanation (A1) divided by corneal displacement. Stiffness parameter A1 uses displacement between the undeformed cornea and A1 and stiffness parameter highest concavity (HC) uses displacement from A1 to maximum deflection during HC [35].

Loss of structural integrity could initiate the reduction in biomechanical properties in keratoconus patients. Subsequently, the focal area will strain more significantly than the surrounding normal area under the same intraocular pressure. The results in thinning of the cornea, further decreasing its biomechanical properties, and further thinning. The outcomes support this hypothesis, describing reduced corneal biomechanical stability before the alteration of corneal shape. SP-A1 could be a potential biomarker evaluating the progression of keratoconus [36].

There are limitations to this study. The main limitation is the paucity of the sample; thus, oversampling techniques must be used. New, more extensive, multicenter studies would help to contrast the results.

## 5. Conclusions

The random forest model is a good model for the classification of subclinical keratoconus. The SP A1 variable turns out to be the most important and determinant in classifying and identifying subclinical keratoconus, followed by A2 Time.

## Figures and Tables

**Table 1:** Demographics and clinical data.

		Mean	Std. Deviation	Std. Error	Sig.
Age(years)	Normal	45.85	20.04	2.57	0.09
	SCKC	40.21	13.19	2.95	
BSCVA (decimal scale)	Normal	0.98	0.05	0.01	0.97
	SCKC	0.99	0.07	0.02	
Sph eq (diopters)	Normal	−1.04	3.16	0.47	0.52
	SCKC	−1.85	1.6	0.44	
KMAX (diopters)	Normal	45.54	2.07	0.27	0.61
	SCKC	46.15	2.12	0.47	
CCT (µm)	Normal	529.48	51.08	6.59	0.17
	SCKC	511.4	30.04	6.72	
IOP (mmHg)	Normal	16.19	3.55	0.45	0.00 *
	SCKC	13.61	2.06	0.46	
HOA RMS (µm)	Normal	0.51	0.25	0.03	0.31
	SCKC	0.63	0.3	0.07	

* *p* < 0.05. STd Deviation= Standard Deviation, BSCVA= Best Corrected Visual Acuity, Sph eq = spherical equivalent = spheric refraction + 1/2 cylinder refraction; KMAX= Keratometry, CCT: Centrl Corneal Thickness, IOP= Intraocular Pressure, HOA RMS= High Order Aberrations.

**Table 2:** Corvis corneal biomechanical data.

		Mean	Std. Deviation	Std. Error	*p*-Value
Def. Amp. Max (mm)	Normal	1.03	0.1	0.01	0.01 *
	SCKC	1.13	0.11	0.02	
A1 Time (ms)	Normal	7.52	0.39	0.05	0.00 *
	SCKC	7.22	0.22	0.05	
A1 Deflection Length(mm)	Normal	2.35	0.31	0.04	0.24
	SCKC	2.24	0.24	0.05	
A1 Velocity (m/s)	Normal	0.14	0.02	0	0.00 *
	SCKC	0.16	0.02	0	
A2 Time (ms)	Normal	21.6	1.03	0.13	0.08
	SCKC	22.01	0.58	0.13	
A2 Deflection Length (mm)	Normal	3.21	0.8	0.11	0.68
	SCKC	3	0.76	0.17	
A2 Velocity (m/s)	Normal	−0.23	0.04	0.01	0.00 *
	SCKC	−0.27	0.04	0.01	
HC Time (ms)	Normal	17.03	0.66	0.09	0.88
	SCKC	17.12	0.4	0.09	
Peak Dist. (mm)	Normal	4.8	0.39	0.05	0.00 *
	SCKC	5.1	0.25	0.06	
Radius (mm)	Normal	7.11	1.18	0.15	0.34
	SCKC	6.75	0.78	0.18	
DA Ratio Max (2 mm)	Normal	4.61	3.46	0.44	1
	SCKC	4.6	0.5	0.11	
DA Ratio Max (1 mm)	Normal	1.68	0.65	0.08	0.8
	SCKC	1.61	0.06	0.01	
Integrated Radius (mm^−1^)	Normal	8.27	1.38	0.18	0.1
	SCKC	9.04	1.34	0.3	
ARTh	Normal	481.87	189.43	24.46	0.00 *
	SCKC	332.46	67.37	15.46	
SP A1	Normal	113.54	18.51	2.39	0.00 *
	SCKC	89.61	15.25	3.41	
CBI	Normal	0.27	0.32	0.04	0.01 *
	SCKC	0.59	0.38	0.09	

* *p* < 0.05. Def Amp Max= Deformation Amplitude Max, Air pulse on (A1time, A1 length and A1 velocity) and off (A2 time, A2 length and A2 velocity), time of highest concavity (time HC), maximum deformation amplitude (DA max), peak distance (PD) and radius of curvature (RHC) at the highest concavity (HC). Vinciguerra Indexes: maximum deformation amplitude radius (DARatio Max) at 2 and 1 mm, integrated radius, relative thickness of Ambrósio in the horizontal profile (ARTh) and stiffness parameter at the first flattening (SP-A1), CBI= Corvis Biomechanical Index.

**Table 3:** Decision Tree. Confusion matrix. Prediction vs reality.

	NORMAL	SCKC
NORMAL	7	1
SCK	7	13

**Table 4:** Decision tree. Metrics of the model.

	Value
Sensitivity	0.50
Specificity	0.93
Pos Pred Value	0.88
Neg Pred Value	0.65
Precision	0.88
Recall	0.50
F1	0.64
Prevalence	0.50
Detection Rate	0.25
Detection Prevalence	0.29
Balanced Accuracy	0.71

**Table 5:** Random Forest: confusion matrix prediction vs. reality.

	NORMAL	SCK
NORMAL	12	1
SCKC	2	13

**Table 6:** Random forest: metrics values.

	Value
Sensitivity	0.86
Specificity	0.93
Pos Pred Value	0.92
Neg Pred Value	0.87
Precision	0.92
Recall	0.86
F1	0.89
Prevalence	0.50
Detection Rate	0.43
Detection Prevalence	0.46
Balanced Accuracy	0.89

## Data Availability

The data presented in this study are available on request from the corresponding author.
